# The Development of Spatial Representation Through Teaching Block-Building in Kindergartners

**DOI:** 10.3389/fpsyg.2020.565723

**Published:** 2020-10-02

**Authors:** Liman Cai, Jiutong Luo, Hui Zhang, Jinling Ying

**Affiliations:** ^1^School of Education, South China Normal University, Guangzhou, China; ^2^Advanced Innovation Center for Future Education, Faculty of Education, Beijing Normal University, Beijing, China; ^3^Faculty of Education, The University of Hong Kong, Pokfulam, Hong Kong; ^4^Teacher’s College, Shihezi University, Shihezi, China

**Keywords:** children, block-building, spatial representation, linguistic representation, graphic representation, model representation

## Abstract

This study investigated the effects of the teaching block-building intervention on overall spatial representation and its three sub-forms, namely linguistic, graphic and model representations, in kindergartners. Eighty-four children (39 girls and 45 boys), aged 5–6 years old, were randomly selected and equally divided into two groups, i.e., experimental group and control group. The experimental group received the intervention of teaching block-building for 14 weeks (45 min each time, once a week), while children in the control group freely played with blocks for the equivalent time. Children’s spatial representation performances were measured in both pre- and post-tests by the *Experimental Tasks of Spatial Representation for Children*. The results showed that: (1) teaching block-building could promote not only the overall spatial representation but also all three sub-forms of spatial representations; (2) there was no gender differences regarding the effect of teaching block-building on neither the overall nor three sub-forms of spatial representations; (3) after the intervention, the diversity of children’s choices regarding the use of sub-forms spatial representations was also promoted in the experimental group. In summary, these results contributed to a comprehensive and systematic understanding of the effects of teaching block-building on spatial representation among children in kindergartens.

## Introduction

Spatial representation, or cognitive representation of spatial relations, refers to how the knowledge of space is represented in the brain (Olson and Bialystok, 1983; Bisiach et al., 1985; Eilan et al., 1993; Grieves and Jeffery, 2017). It belongs to a broad concept known as spatial ability or spatial skills. Generally, spatial ability refers to the capacity for individuals to generate, retain, retrieve, and transform well-structured information, such as visual, diagrammatic, or symbolic form (Lohman et al., 1987; Lohman, 1996). It may involve not only the understanding of the outside world but also the processing of outside information and reasoning with it through representation in mind (Kosslyn, 1995), i.e., spatial representation.

Spatial ability is vital for achievement in subjects and careers related to Science, Technology, Engineering, and Mathematics (i.e., STEM) (Caldera et al., 1999; Assel et al., 2003; Chen, 2009; Wai et al., 2009; Uttal and Cohen, 2012; Stieff and Uttal, 2015; Ha and Fang, 2016). The claim that spatial ability contributes to STEM academic performance has been verified in many subjects, such as chemistry, physics, and anatomy, etc. (e.g., Pallrand and Seeber, 1984; Delialioğlu and Aşkar, 1999; Harle and Towns, 2011; Lufler et al., 2012; Sweeney et al., 2014). In recent years, STEM has been emphasized in all periods of school education, as well as early childhood education; hence, it is also of great importance to pay attention to the development of children’s spatial ability.

### Previous Studies of Teaching Block-Building on Spatial Ability

Following this direction, it has been suggested that children engage in building and playing with blocks could significantly promote their spatial ability (e.g., Brosnan, 1998; Caldera et al., 1999; Martin-Dorta et al., 2014; Verdine et al., 2014a,b; Jirout and Newcombe, 2015). For example, Casey et al. (2008) reported that block-building supported the development of spatial visualization in kindergartners. Traditionally, there are two main aspects of spatial ability found to be closely related to block-building activities–spatial visualization and mental rotation (Linn and Petersen, 1985; Casey et al., 2008; Newman et al., 2016). On the one hand, spatial visualization, which involves the ability to generate images of different shapes and then mentally combine them to produce a new design, is necessary for all block building activities (Newman et al., 2016). It is suggested that when a child is playing with blocks, he or she is mentally visualizing how blocks will fit and interact with one another (Newman et al., 2016); therefore, building blocks may facilitate the development of spatial visualization. On the other hand, mental rotation, which consists of the ability to look at an object or a picture of an object and visualizes what it might look like if rotated in either two- or three-dimensional space, has also been commonly found during block building activities (Casey et al., 2008). That is, children will inevitably utilize strategies to rotate blocks to different orientations and build the whole structure. For example, when building blocks, children do spatial flips to fit them into a particular slot in the structure, and spatial turns to make corners with blocks. In this case, children’s block-building activities may generate many benefits to their spatial ability development. Moreover, this benefit on spatial ability, which comes from block-building and similar activities, is even higher than children could gain from other usually played activities, such as drawing, playing with sound-producing toys (e.g., guitars) and other toys (e.g., trucks), riding bikes, etc. (Jirout and Newcombe, 2015).

Although researchers have dedicated to investigating how block-building could contribute to children’s spatial ability, few of their efforts have been invested to directly test whether and how block-building can benefit children’s spatial representation specifically. In particular, there are different sub-forms of spatial representations. For example, researchers have identified that there are linguistic and non-linguistic categorizations of spatial relations (Hayward and Tarr, 1995; Crawford et al., 2000). In terms of the non-linguistic aspect, it has been suggested that spatial representation also contains the cognitive representation of spatial relations on a map (Kulhavy et al., 1983), which is also called map representation (Blaut and Stea, 1971; Landau, 1986), including both two- and three-dimensional forms (i.e., graphic and model, respectively). Researchers have highlighted the importance of these sub-forms of spatial representations, i.e., linguistic, graphic, and model, during early childhood development (Pang et al., 2008). For this study, we briefly propose the executive definitions of these sub-forms according to the previous literature (e.g., Blaut and Stea, 1971; Bluestein and Acredolo, 1979; Landau, 1986; DeLoache, 1989, 2000; Landau and Jackendoff, 1993; Netelenbos and Savelsbergh, 2003; Szechter and Liben, 2004; Pang et al., 2008; Lahav et al., 2018). First, linguistic representation is the ability to express spatial experience and relations through language. Second, graphic representation, also called map-reading skills, means children’s ability to infer the position of an object in a three-dimensional environment from information contained on a two-dimensional map. Third, model representation refers to the self-orientation, object-orientation, and place-orientation of the three-dimensional object in the real environment according to the model; and the model is used as a symbol for obtaining information about the position of the object in the real world. Similar to the overall spatial representation, there is also a lack of research on whether and how block-building can improve these sub-forms of spatial representations among children during early childhood. In this case, it is needed to design intervention studies that are focusing on exploring whether block-building can benefit the development of spatial representation, including its sub-forms. Therefore, in the following sections, we first depict how block-building could relate to the development of spatial representation. Then, the potential influence factors to intervention effects, in particular gender, are also discussed.

### Why and How Can Block-Building Contribute to Spatial Representation?

It is regarded as a significant improvement of research efforts to shift to explore how children use spatial representation to understand the real world. It is evidenced that block-building can help children improve their perception of spatial relations in the real world, i.e., overall spatial representation. For example, through the analysis of practical teaching cases, researchers have found that block-building may help children understand the concept of orientation and improve their cognition of spatial relations (Blades and Cooke, 1994).

Moreover, there are also pieces of evidence that block-building could benefit the sub-forms of spatial representations among children. First, block-building can improve children’s language use regarding space, i.e., linguistic representation. For example, some scholars have tested to provide children with a complete three-dimensional bonding model and a set of randomly arranged blocks and ask them whether the blocks fit the model. Results have indicated that block-building promoted the communication using spatial language between children and adults (Verdine et al., 2014a,b). Another experimental study also found that teachers’ use of spatial language during teaching block-building would further encourage children to use more spatial vocabulary as well (Cohen and Emmons, 2017); therefore, it could further improve children’s linguistic representation. Second, block-building that also brings challenges to children’s graphic cognition may also be conducive to their spatial ability (Szechter and Liben, 2004), especially the graphic representation. For example, a qualitative study has found that block-building activity is beneficial to children’s understanding of geometric shapes (Park et al., 2008); thus it might facilitate children’s representation ability in two-dimensional aspect. Therefore, it would contribute to the development of graphic representation, as well. Finally, block-building involves enriched interactions with three-dimensional objects; in this case, it will also possibly improve children’s model representation of space. Based on the above literature review, we accordingly propose that block-building is a promising activity for improving children’s spatial representation, which might include not only the overall spatial representation but also its sub-forms.

### Will Gender Be an Influential Factor in the Intervention Effect?

Recently, various studies have also been conducted to identify the factors that might affect children’s spatial representation. It has been suggested that individual experiences, e.g., family social-economic status (SES), construction materials, etc., are all potential factors that may influence the development of spatial ability among children (e.g., Caldera et al., 1999; Verdine et al., 2014a). Among these factors, gender is one of the most frequently mentioned variables (e.g., Casey et al., 2008; Jirout and Newcombe, 2015). Some scholars have reported that boys usually outperform girls on some spatial tasks (e.g., Casey et al., 1995), and are better at mastering building skills and understanding the structure balance with blocks (Tian et al., 2018). However, there are also inconsistent findings regarding this gender difference. For example, a longitudinal study has shown that there is no significant gender difference in spatial ability after 3 years of equal time playing with blocks (Hanline et al., 2001). Therefore, it remains unclear whether there are gender differences regarding the development of children’s spatial representation with the intervention of block-building.

### Research Gaps

To sum up, researchers have invested considerable efforts to explore the effects of block-building on the development of spatial ability, as well as spatial representation, and have obtained significant research findings. However, there are still research gaps that need to be filled. First, spatial representation consists of at least three sub-forms: linguistic, graphic, and model. Most previous studies have studied these sub-forms of spatial representations separately (e.g., Szechter and Liben, 2004; Ferrara et al., 2011); however, few of them have investigated them comprehensively and explored how these sub-forms of spatial representations can be promoted by the block-building within the same intervention or training program. The *Experimental Tasks of Spatial Representation for Children* developed by Pang et al. (2008) is among the very few tasks that can test spatial representations regarding its sub-forms among Chinese children. Therefore, this study aims to use this task to examine how children’s spatial representation and its sub-forms can be promoted by the intervention of block-building more comprehensively and systematically. Second, previous training or intervention programs are relatively independent of the conventional teaching processes of kindergarten (e.g., Newman et al., 2016). There are also very few studies that have investigated to what extent a systematic instruction of block-building by teachers or assistants could benefit children’s spatial representation. The intervention of teaching block-building, which will be used in this study, refers to an instructional process that teachers plan and organize the symbolic and constructive gaming activities according to the teaching objectives and contents. In addition, within this kind of systematic instruction, whether gender differences in children’s spatial representation could exist needs to be further explored. Last but not least, most of the previous studies have focused on the development of spatial abilities; thus, it is still unclear that when children exposure to a new task, how would they choose to finish it with different forms of spatial representations. Therefore, children’s choices regarding these three sub-forms of spatial representations should also be investigated.

### The Present Study

To fill the research gaps mentioned above, we use the experimental design, including both experimental and control groups, to explore whether the intervention of teaching block-building could benefit spatial representation and its sub-forms in a kindergarten. The research questions (RQs) are specified as followings:

RQ1: Will the intervention of teaching block-building improve children’s overall spatial representation while considering gender?
RQ2: Will the intervention of teaching block-building benefit children’s sub-forms of spatial representations, i.e., linguistic, graphic, and model, respectively, while considering gender?
RQ3: How will children use their different spatial representations? That is, will children receiving the intervention use more sub-forms of spatial representations in a given context?

## Materials and Methods

### Participants

A total of 84 children, aged 5–6 years old, were randomly selected from a kindergarten in a city of southern China and were equally divided into two groups, i.e., experimental group (42 children, with 20 girls and 22 boys) and control group (42 children, with 19 girls and 23 boys). We did not take an additional screening process on the development conditions of children for two reasons. On the one hand, Chinese kindergartens have not to implement inclusive education at the moment. On the other hand, according to the teacher, no extra care should be paid to children in both groups. Therefore, children should be regarded as typically developed from the selected kindergarten. The Chi-square test revealed that there were no differences in gender between two groups (χ^2^ = 0.048, *p* = 0.827). The principal of the kindergarten and all teachers agreed to participate before the recruitment of participants. Parental approval and children’s consent were also obtained before the study. This study was approved by the ethics review committee at the first author’s institute.

### Measures

**FIGURE 1:**
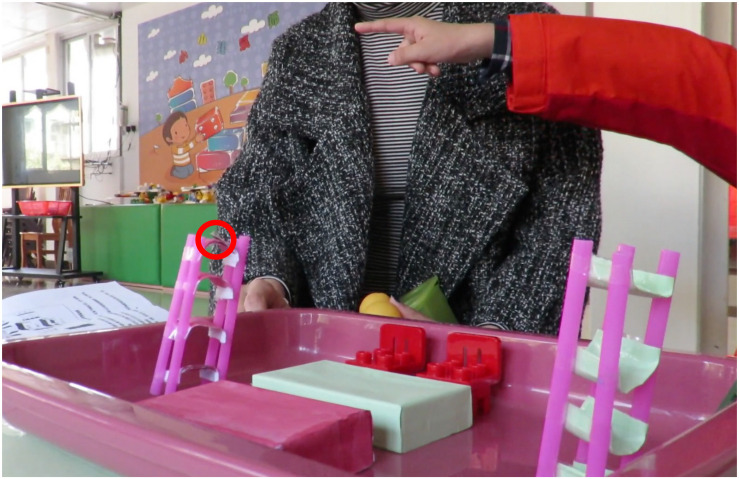
A sample task from the Experimental Tasks of Spatial Representation for Children.

Children’s spatial representation was tested by the *Experimental Tasks of Spatial Representation for Children* (Pang et al., 2008). This test was developed by a team of expert Chinese scholars in early childhood development and was supported with face validity in early studies (Pang et al., 2008). Four games (or sub-tasks) were included in the test: *taking the bear home, hide and seek (I)*, *hide and seek (II)*, and *hiding treasures*. First, the game *taking the bear home*, in which participants should place items as required, focused on the development of children’s linguistic representation. Second, the game *hide and seek (I)*, in which the participants were asked to find the target location according to the model and then describe the location in their own words, with a focus on investigating their abilities of linguistic and model representations. Third, the game *hide and seek (II)*, in which children should find the targets by referring to picture cards and then orally describe the target locations, which was designed to test children’s performance of linguistic and graphic representations. Last, the game *hiding treasures*, where children were encouraged to use appropriate forms of spatial representation to tell others the spatial information of an object in a free context, was intended to examine the development of children’s linguistic, graphic and spatial representations, as well as the comprehensive use of these sub-forms of spatial representations. For an easy understanding, here one sample task, i.e., hide and seek (I), was shown. As shown in Figure 1, the researcher used materials to construct a room represent the model of the room and there was a red circle represents there was something hide there. Children were asked to indicate the target location in the real room accordingly and then describe the location in their own words. Therefore, this task tested both linguistic and model representations.

According to the tasks above, the total score for the whole test consisted of the scores from the sub-scores of spatial representations: linguistic, graphic, and model representations. The total score of the test was 64 points, of which linguistic representation accounted for 16 points (8 scoring points), and graphic and model representations both accounting for 24 points (12 scoring points), respectively. On each scoring point, children were rated on a three-point scale (i.e., 0–2) and then the results were summarized into a total score for each sub-form of spatial representation. For example, when testing the graphic representation, a child would get zero points if he or she failed to identify the target location, get one point if he or she referred to the nearby location, and get two points if he or she identified the target location precisely. As for the comprehensive use of spatial representation, it referred to how many types of spatial representations children could use to finish the last task. The score was ranged from 1 (i.e., using one sub-form only) to 3 (i.e., using all three sub-forms). This score also reflected children’s ability in the diversity of choices regarding the use of sub-forms spatial representations.

### Experimental Design and Procedure

The pre- and post-test experimental design was adopted to investigate the effects of teaching block-building on children’s spatial representation. The experimental group received the teaching block-building intervention (45 min each time, once a week) for 14 weeks, while no intervention was applied to the control group’s block-building activities during this period. Both groups also had the same other activities in the kindergarten and were required not to play blocks after school during the whole period.

Two themes of block-building activities, i.e., “*I Love You, My Motherland*” and “*I Am Here for Emergency Help*,” were used as teaching materials for the experimental group. Within each theme, both object and context constructions were included. Each of the theme took about 7 weeks and contained about five or six object construction sessions, such as helicopter, ambulance, fire truck etc., and one to two content construction sessions. Educational activities were generally made up of four steps: first, children should observe both physical and block modeling pictures to understand the structure of the object (object observation); second, children explored ways to represent characteristics of the object with different blocks (exploration and analysis); third, children used various blocks to build the object (free construction); and last, children shared their constructed works and ideas with others (sharing and expression). For example, children were asked to build an ambulance in one object construction session. In the first step, children were shown pictures of the ambulance, which displaying its front, rear, left, right, internal, and outward appearances, and they observe them carefully. Then, children explored which blocks they could use to build the ambulance. Later on, they freely construct the different parts of the ambulance and combine them. And finally, they shared with others about their thoughts and processes. The teaching activities were performed by a teacher from the participating kindergarten. The main role of the teacher was to observe during children’s block-building and identify difficulties that children might face to help them proceed. They did not directly teach or model block-building to children. And also, spatial language was not intentionally taught to children, however, it could happen naturally in some circumstances, such as general introduction or problem solving.

The assessment was conducted by well-trained postgraduates, who majored in early childhood education. The students who performed the assessment were blinded of children’s group conditions during the testing process. The pre- and post-tests were implemented individually. Before each test, experimenters were asked to begin with a pilot test to adjust their guidance. Then experimenters were required to take participants to warm up and helped them get familiar with the experimental environment and materials. The formal test was conducted strictly following the unified instruction, and experimenters were randomly assigned to record children’s answers. The first three tasks and the fourth task were separated by an interval of 2–3 days to avoid potential interferences. When the tests were completed, children’s performances were coded according to the recording sheet and were input to SPSS for analysis.

## Results

A series of statistical analyses, including descriptive analysis, mixed ANOVAs, and Chi-square test, were performed on the collected data. Table 1 shows both the mean (SD) from descriptive analysis and adjusted mean (SE) from mixed ANOVAs of overall and sub-forms of spatial representations for both groups.

**TABLE 1:** Mean (SD) and adjusted mean (SE) of overall and sub-forms of spatial representation by group and time.

Measures	Group	Pre-test		Post-test	
			
		Mean (SD)	Adjusted mean (SE)	Mean (SD)	Adjusted mean (SE)
Overall spatial representation	Experimental	46.36 (4.62)	46.35 (0.74)	52.74 (3.39)	52.74 (0.64)
	Control	46.14 (4.87)	46.08 (0.74)	46.21 (4.79)	46.15 (0.65)
Linguistic representation	Experimental	10.86 (1.88)	10.88 (0.28)	12.98 (1.42)	13.00 (0.24)
	Control	10.29 (1.76)	10.26 (0.28)	10.38 (1.68)	10.36 (0.24)
Graphic representation	Experimental	16.62 (2.27)	16.60 (0.36)	19.00 (1.99)	18.99 (0.33)
	Control	17.40 (2.32)	17.38 (0.36)	17.67 (2.28)	17.61 (0.33)
Model representation	Experimental	18.88 (2.76)	18.88 (0.40)	20.76 (2.06)	20.76 (0.34)
	Control	18.45 (2.34)	18.44 (0.40)	18.74 (2.30)	18.73 (0.34)

### Effects on Children’s Overall Spatial Representation

To explore whether the teaching block-building can generate different effects on overall spatial representation and whether there are differences between girls and boys, we carried out a 2 (Gender: boys and girls) × 2 (Group: experimental group and control group) × 2 (Time: pre- and post-tests) mixed ANOVA. The results showed that the interactions between group, gender and time were not significant [*F*(1,80) = 0.267, *p* = 0.607 > 0.05, $\eta_{\text{p}}^{2}$ = 0.003], nor was the interaction between gender and time [*F*(1,80) = 0.073, *p* = 0.787 > 0.05, $\eta_{\text{p}}^{2}$ = 0.003]. Besides, there were no main effects of gender as well [*F*(1,80) = 0.532, *p* = 0.468 > 0.05, $\eta_{\text{p}}^{2}$ = 0.007]. These results suggested that after the intervention of teaching block-building, there was no gender difference between the experimental group and the control group.

**FIGURE 2:**
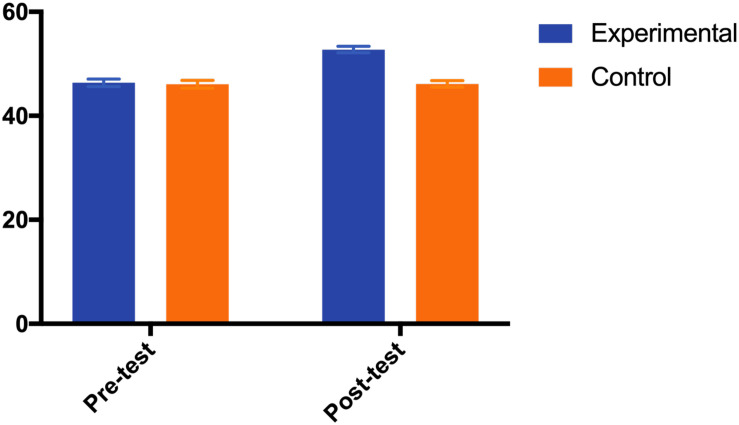
Mean for overall spatial representation by test and group.

However, the interaction between group and time was significant [*F*(1,80) = 142.012, *p* = 0.000, $\eta_{\text{p}}^{2}$ = 0.640] and the main effect of time [*F*(1,80) = 147.929, *p* = 0.000, $\eta_{\text{p}}^{2}$ = 0.649] and group [*F*(1,80) = 13.212, *p* = 0.000, $\eta_{\text{p}}^{2}$ = 0.142] were both significant. As a result, it suggested that the intervention had a significant promoting effect on children’s overall spatial representation. The *post hoc* analysis revealed that, regarding the overall spatial representation, while children in the control group maintained their performance [Adjusted Mean_pre_ (SE) = 46.08 (0.74), Adjusted Mean_post_ (SE) = 46.15 (0.65); *p* = 0.863], children’s performance in experimental group was significantly improved [Adjusted Mean_pre_ (SE) = 46.35 (0.74), Adjusted Mean_post_ (SE) = 52.74 (0.64); *p* = 0.000]. The results are shown in Figure 2.

### Effects on Different Sub-Forms of Spatial Representation

To investigate the effects of teaching block-building on sub-forms of spatial representations, i.e., linguistic, graphic, and model, respectively, and whether there are gender differences between girls and boys. We also performed three 2 (Gender: boys and girls) × 2 (Group: experimental group and control group) × 2 (Time: pre- and post-tests) mixed ANOVAs in this section. The results are shown in the following sections.

### The Effect on Linguistic Representation

Regarding the linguistic representation, the results showed that the interactions between group, gender and time were not significant [*F*(1,80) = 0.015, *p* = 0.904 > 0.05, $\eta_{\text{p}}^{2}$ = 0.000], nor was the interaction between gender and time [*F*(1,80) = 0.143, *p* = 0.706 > 0.05, $\eta_{\text{p}}^{2}$ = 0.002]. Besides, there were no main effects of gender as well [*F*(1,80) = 0.347, *p* = 0.558 > 0.05, $\eta_{\text{p}}^{2}$ = 0.004]. These results suggested that after the intervention of teaching block-building, there was no gender difference between the experimental group and the control group on linguistic representation.

**FIGURE 3:**
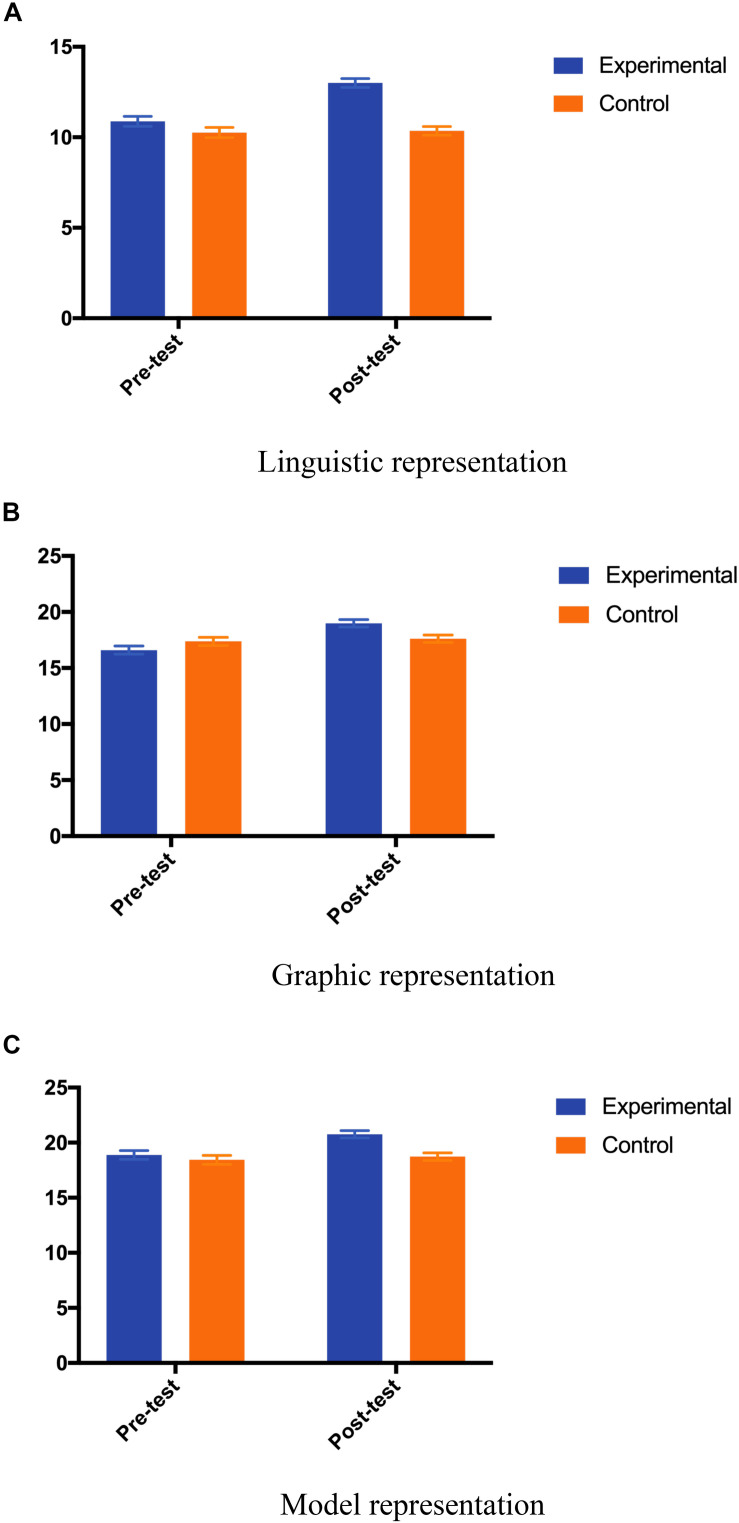
Mean for sub-forms of spatial representations by test and group. **(A)** Linguistic representation. **(B)** Graphic representation. **(C)** Model representation. The *y*-axis is not the same across there sub-figures.

Besides, the results also showed that the interaction between group and time was significant [*F*(1,80) = 77.57, *p* = 0.000, $\eta_{\text{p}}^{2}$ = 0.492] with the main effect of time [*F*(1,80) = 93.81, *p* = 0.000, $\eta_{\text{p}}^{2}$ = 0.540] and group [*F*(1,80) = 21.761, *p* = 0.000, $\eta_{\text{p}}^{2}$ = 0.214] were both significant. As a result, it suggested that the intervention had a significant promoting effect on children’s linguistic representation. The *post hoc* analysis revealed that, regarding the linguistic representation, while children in the control group maintained their performance [Adjusted Mean_pre_ (SE) = 10.26 (0.28), Adjusted Mean_post_ (SE) = 10.36 (0.24); *p* = 0.553], children’s performance in experimental group was significantly improved [Adjusted Mean_pre_ (SE) = 10.88 (0.28), Adjusted Mean_post_ (SE) = 13.00 (0.24); *p* = 0.000]. The results are shown in Figure 3A.

### The Effect on the Graphic Representation

Similarly, the results on graphic representation showed that the that the interactions between group, gender and time were not significant [*F*(1,80) = 1.477, *p* = 0.228 > 0.05, $\eta_{\text{p}}^{2}$ = 0.018], nor was the interaction between gender and time [*F*(1,80) = 0.116, *p* = 0.734 > 0.05, $\eta_{\text{p}}^{2}$ = 0.001]. Besides, there were no main effects of gender as well [*F*(1,80) = 2.711, *p* = 0.104 > 0.05, $\eta_{\text{p}}^{2}$ = 0.033]. These results suggested that after the intervention of teaching block-building, there was no gender difference between the experimental group and the control group on graphic representation.

Furthermore, the interaction between group and time was significant [*F*(1,80) = 34.070, *p* = 0.000, $\eta_{\text{p}}^{2}$ = 0.299] with the main effect of time also significant [*F*(1,80) = 50.526, *p* = 0.000, $\eta_{\text{p}}^{2}$ = 0.387]. As a result, it suggested that the intervention had a significant promoting effect on children’s graphic representation. The *post hoc* analysis revealed that, regarding the graphic representation, while children in the control group maintained their performance [Adjusted Mean_pre_ (SE) = 17.38 (0.36), Adjusted Mean_post_ (SE) = 17.61 (0.33); *p* = 0.316], children’s performance in experimental group was significantly improved [Adjusted Mean_pre_ (SE) = 16.62 (0.35), Adjusted Mean_post_ (SE) = 19.00 (0.33); *p* = 0.000]. The results are shown in Figure 3B.

### The Effect on Model Representation

Finally, the results on model representation showed that the interactions between group, gender and time were not significant [*F*(1,80) = 001, *p* = 0.977 > 0.05, $\eta_{\text{p}}^{2}$ = 0.000], nor was the interaction between gender and time [*F*(1,80) = 0.019, *p* = 0.889 > 0.05, $\eta_{\text{p}}^{2}$ = 0.000]. Besides, there were no main effects of gender as well [*F*(1,80) = 0.206, *p* = 0.651 > 0.05, $\eta_{\text{p}}^{2}$ = 0.003]. These results suggested that after the intervention of teaching block-building, there was no gender difference between the experimental group and the control group on model representation.

In addition, the interaction between group and time was significant [*F*(1,80) = 23.705, *p* = 0.000, $\eta_{\text{p}}^{2}$ = 0.229] with the main effect of time [*F*(1,80) = 43.967, *p* = 0.000, $\eta_{\text{p}}^{2}$ = 0.355] and group [*F*(1,80) = 6.066, *p* = 0.016, $\eta_{\text{p}}^{2}$ = 0.070] were both significant. As a result, it suggested that the intervention had a significant promoting effect on children’s model representation. The *post hoc* analysis revealed that, regarding the model representation, while children in the control group maintained their performance [Adjusted Mean_pre_ (SE) = 18.45 (0.40), Adjusted Mean_post_ (SE) = 18.74 (0.34); *p* = 0.214], children’s performance in experimental group was significantly improved [Adjusted Mean_pre_ (SE) = 18.88 (0.40), Adjusted Mean_post_ (SE) = 20.76 (0.34); *p* = 0.000]. The results are shown in Figure 3C.

### The Effect on Children’s Diversity of Choices Regarding Spatial Representations

We also conducted an additional statistical analysis regarding the children’s choices of sub-forms spatial representations in a free context where they could construct an object with blocks. In the pre-test, the proportion of children in the experimental group who chose to use one representation form and two or more representation forms were 83 and 17%, respectively. In contrast, the proportions for children in the control group were 86 and 14%, respectively. After the intervention, in the experimental group, those who chose to use one spatial representation form and those who chose to use two or more representation forms came up to an equal proportion, both accounting for 50%. However, in the control group, 76% of children still decided to use one spatial representation form, while only 24% of children chose more than two or more spatial representation forms.

**FIGURE 4:**
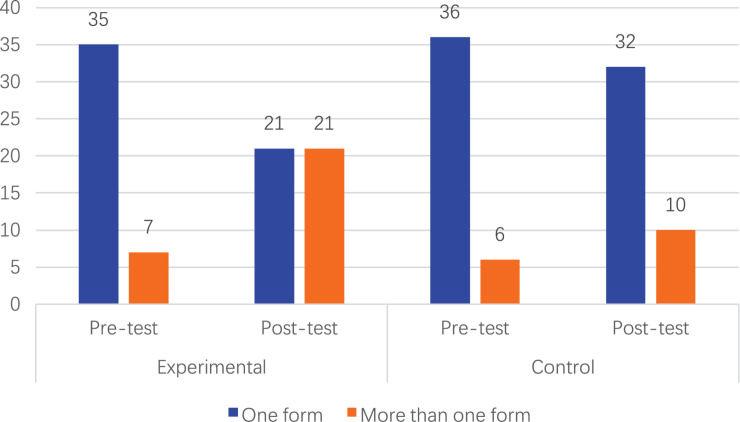
Children’s diversity of choices regarding sub-forms of spatial representations.

It was identified in Figure 4 that the number of children in the experimental group who chose to use two or more spatial representation forms increased significantly after the intervention (χ^2^ = 10.500, *p* = 0.001 < 0.01). By contrast, in the control group, more children still chose only to use one spatial representation form than those who used two or more spatial representation forms in both pre- and post-tests (χ^2^ = 1.235, *p* = 0.266 > 0.05). The results revealed that after the intervention, children tended to diversify their choices in using more sub-forms of spatial representations.

## Discussion

### Main Effects of Teaching Block-Building on Children’s Spatial Representations

This study investigated the effects of teaching block-building on children’s spatial representation, which was measured by the *Experimental Tasks of Spatial Representation for Children* by Pang et al. (2008), employing the experimental design. By comparing data collected from both control and experimental groups, the effects of teaching block-building on the overall spatial representation were supported. We further confirmed that it was due to the improvements on all three sub-forms of spatial representations, i.e., linguistic, graphic, and model representations, respectively.

Firstly, this study found that teaching block-building promoted the development of children’s linguistic representation. There are mainly three possible reasons for this result. First of all, compared with non-spatial games, block-building, as a common spatial activity, involves more spatial language (Verdine et al., 2014a,b). Children take spatial features, such as object characteristics (e.g., large, small, tall, short, etc.), shapes (e.g., circle, square, triangle, etc.) and spatial attributes of objects (e.g., bending, edge, etc.), as the communication contents in the game (e.g., Hanline et al., 2001). In this case, the spatial language has been involved in nearly all the block-building activities; therefore, it could enhance children’s linguistic representation regarding space. Besides, in the process of analyzing the structure of constructions and exploring the construction method, teachers provided children with numbers, sizes, locations, space distances, and model scale and structure. For example, children were required to listen carefully to and understand the vocabulary, and to grasp spatial information to complete the construction of target objects. Accordingly, the representation system provided by spatial language could explain equivalent spatial concepts. For example, Sun (2005) found that children could understand the relationship between spatial language and space in the real world. When teachers guided them to observe the characteristics of construction objects using words, they provided spatial information to children, such as “put on the top of the cabin,” “set on the tail behind the wings,” and so on. Therefore, these verbal instructions would have provided children with a rich experience of linguistic representation; thus, it could improve it accordingly.

Moreover, the sharing and communicating section were also included as an essential part of teaching block-building. It encouraged children to express their ideas freely. Therefore, children might have opportunities to use spatial language to express the construction process and introduce their works. In this case, the frequency of using spatial language among children, as well as between children and teachers, could distinctly increase during the teaching of block-building, which might further contribute to the development of children’s linguistic representation. This explanation was also supported by the experiment result that block-building could stimulate children to use more spatial language, and therefore, to promote their development of linguistic representation (Ferrara et al., 2011).

Secondly, teaching block-building also promoted the ability of children’s graphic representation. Two points might contribute to this effect. On the one hand, the use of images during the block-play games would contribute to children’s graphic representation. It was suggested that spatial relationships within graphic symbols represented the real relationships between the actual objects (Bluestein and Acredolo, 1979). In our study, teachers might ask children to observe the images of the objects for construction and the pictures of various block models from which children could extract information of object features into their minds. Then children used the blocks to construct the object freely. For example, when required to build an ambulance in the second game, children were shown by the teacher pictures of the ambulance, which displaying its front, rear, left, right, internal, and outward appearances. Then children participated in observation, meditation, and discussion for which blocks should be chosen to construct these different parts of the ambulance. Therefore, graphic representation would be cultivated through the use of images. On the other hand, some activities in the teaching process might also benefit graphic representation. For instance, children also tried to implement their thoughts by skills, such as tiling, bridging, dislocating, and enclosing after seeing pictures of real objects and complete works. This process was designed to enable children to think, reason, and operate on spatial information (Pang et al., 2008), and could promote the development of children’s graphic representation as well.

Thirdly, teaching block-building promoted the development of children’s model representation. Pang et al. (2008) suggested that model representation also belonged to the map spatial representation. Therefore, to fully understand the spatial representation of maps, children must understand the spatial and geometric correspondence between representing objects and referent objects in terms of distance, perspective, and orientation. In this case, the relationship between models and real-world objects was also in correspondence with a spatial relationship in blocks. In this study, the spatial factors included in the teaching block-building activities could provide children with a variety of spatial concepts and spatial relations. Moreover, children in the experimental group also had more opportunities to understand the position of each part of the block-building and the relationship among each section, and to relate it to objects in the real world. Thus, it supported the finding that teaching block-building could improve the representation ability of model space with the help of children’s understanding of spatial concepts and spatial relationships, i.e., model representation.

Fourth, this study revealed that there were around six points of improvement on children’s overall spatial representation; and each of the sub-forms contributed around two points after the intervention. This result might suggest that our intervention would be equally effective for all three sub-forms of spatial representations. Therefore, this intervention would be an important reference to future research which interested in improving all these three sub-forms of spatial representations together.

Finally, the gender effect, which might affect children’s spatial representation, was also discussed. It turned out that there was no significant gender difference in neither the overall nor the three sub-forms of children’s spatial representations, which were consistent with the previous result in spatial ability, such as Hanline et al. (2001). However, as noted earlier, studies had reported that boys had advantages over girls in spatial ability (e.g., Tian et al., 2018), which might be regarded as contradictory to the finding of this study. We suspected that this was possible because boys and girls had equal opportunities to play with blocks and were very interested in block-building due to this gamified intervention. And in our study, children in the experimental group spent equal time playing with blocks in the kindergarten. They were also controlled by not allowing them to play with blocks at home during the experiment. In this case, combining with the previous literature, our results also suggested that, if girls were offered a lot of opportunities to play with blocks and their interest in learning was aroused (e.g., with gamified activities), their spatial representation could be developed as well as that of boys.

### The Use of Spatial Representations Will Be Diversified

The results of this study showed that children’s use of spatial representation was more diversified in the experimental group than the control group after the intervention. According to the literature, the development of children’s spatial cognition is a process that activates an individual’s spatial sense from real life to form spatial concepts in their minds (Grieves and Jeffery, 2017). Therefore, when teachers had no requirements on the use of representation forms, the linguistic representation, which is closely related to children’s daily lives, was highly preferred. However, after the intervention, children’s abilities of graphic and model representations were both significantly improved, and the frequency of children’s usage of these two forms of spatial representations could also increase. Moreover, with the accumulation of experience and the gradual development of spatial cognitive ability, children would gradually be able to use a variety of spatial representation forms comprehensively. For example, a child in the pre-test expressed the information of locations of the object only with “here,” but she used more than one form of spatial representation for description in the post-test. She picked up a toy model in front of the experimenter according to the location of physical simulation, then took up picture cards to pose the position, and finally used the sentence “under the table in red, the one on the top of the green table” to deliver the spatial information.

## Conclusion and Limitations

This study is among the very few studies that used teaching block-building as an intervention to comprehensively and systematically investigate the development of children’s spatial representation with experimental design and has gained valuable research findings. First, this study finds that teaching block-building can improve both the overall development of the spatial representation and the three sub-forms of spatial representations: linguistic, graphic, and model. Second, no gender difference has been found, which indicates boys and girls perform equally well with the support of the intervention. Third, the intervention of teaching block-building can also improve the diversity of children’s choices regarding the use of spatial representation forms.

Nevertheless, this study also has some limitations. First, it only randomly selects participants aged 5–6 years old within one selected kindergarten in a city of China, which may limit the generalization ability of the results. Future research should consider recruiting more diverse samples to explore the effects of this teaching block-building intervention on diverse aspects of spatial representations among children. Second, this study only focuses on the spatial representation itself. Future research should consider involving achievement measures of STEM to explore whether the effect could be transferred. Third, only pre- and post-tests are collected in this study; therefore, the longitudinal design may contribute to the understanding of sustaining effects of the intervention in the future. Fourth, what has been used to evaluate the effect on spatial representation is the traditional task only; therefore, methodological innovations, such as integrating neuroimaging methods–electroencephalogram (EEG), functional magnetic resonance imaging (fMRI) and other brain imaging techniques–may contribute a better understanding of children’s spatial representation development and provide a more scientific basis for the effects of this intervention among children.

## Data Availability Statement

The raw data supporting the conclusions of this article will be made available by the authors, without undue reservation.

## Ethics Statement

The studies involving human participants were reviewed and approved by the Ethics Review Committee of the School of Education, South China Normal University. Written informed consent to participate in this study was provided by the participants’ legal guardian/next of kin.

## Author Contributions

LC designed and supervised the research, and wrote and revised the manuscript. JL analyzed the data, and wrote and revised the manuscript. HZ and JY conducted the research, collected the data, and participated in writing the first draft of the manuscript. All authors contributed to the article and approved the submitted version.

## Conflict of Interest

The authors declare that the research was conducted in the absence of any commercial or financial relationships that could be construed as a potential conflict of interest.
